# Postnatal gestational age estimation using newborn screening blood spots: a proposed validation protocol

**DOI:** 10.1136/bmjgh-2017-000365

**Published:** 2017-07-27

**Authors:** Malia S Q Murphy, Steven Hawken, Katherine M Atkinson, Jennifer Milburn, Jesmin Pervin, Courtney Gravett, Jeffrey S A Stringer, Anisur Rahman, Eve Lackritz, Pranesh Chakraborty, Kumanan Wilson

**Affiliations:** 1 Clinical Epidemiology Program, Ottawa Hospital Research Institute, Ottawa, Canada; 2 Newborn Screening Ontario, Children’s Hospital of Eastern Ontario, Ottawa, Canada; 3 Maternal and Child Health Division, International Centre for Diarrhoeal Disease Research, Dhaka, Bangladesh; 4 Global Alliance to Prevent Prematurity and Stillbirth, Seattle, USA; 5 Department of Obstetrics and Gynecology, University of North Carolina at Chapel Hill, Chapel Hill, USA; 6 Matlab Health Research Centre, International Centre for Diarrhoeal Disease Research, Dhaka, Bangladesh

**Keywords:** gestational age, validation study, metabolomics, newborn screening, screening, obstetrics, epidemiology

## Abstract

**Background:**

Knowledge of gestational age (GA) is critical for guiding neonatal care and quantifying regional burdens of preterm birth. In settings where access to ultrasound dating is limited, postnatal estimates are frequently used despite the issues of accuracy associated with postnatal approaches. Newborn metabolic profiles are known to vary by severity of preterm birth. Recent work by our group and others has highlighted the accuracy of postnatal GA estimation algorithms derived from routinely collected newborn screening profiles. This protocol outlines the validation of a GA model originally developed in a North American cohort among international newborn cohorts.

**Methods:**

Our primary objective is to use blood spot samples collected from infants born in Zambia and Bangladesh to evaluate our algorithm’s capacity to correctly classify GA within 1, 2, 3 and 4 weeks. Secondary objectives are to 1) determine the algorithm's accuracy in small-for-gestational-age and large-for-gestational-age infants, 2) determine its ability to correctly discriminate GA of newborns across dichotomous thresholds of preterm birth (≤34 weeks, <37 weeks GA) and 3) compare the relative performance of algorithms derived from newborn screening panels including all available analytes and those restricted to analyte subsets. The study population will consist of infants born to mothers already enrolled in one of two preterm birth cohorts in Lusaka, Zambia, and Matlab, Bangladesh. Dried blood spot samples will be collected and sent for analysis in Ontario, Canada, for model validation.

**Discussion:**

This study will determine the validity of a GA estimation algorithm across ethnically diverse infant populations and assess population specific variations in newborn metabolic profiles.

**Key questions**

**What is already known about this topic?**

- Knowledge of gestational age is critical for guiding neonatal care and quantifying regional burdens of preterm birth. Novel methods of postnatal gestational age estimation are actively being sought.
- We and others have demonstrated that postnatal algorithms developed from newborn metabolic profiles provide gestational age estimates, accurate to within 1–2 weeks.
- Currently published gestational age algorithms have been validated in North American cohorts only.

**What are the new findings?**

- This protocol details an international validation study of a postnatal gestational age algorithm using data collected from newborns born in Zambia and Bangladesh.

## Background

Knowledge of gestational age at the time of birth is important for distinguishing the preterm from the small-for-gestational age (SGA) infant, whose medical needs and expectations for achieving significant development milestones may be different. Reliable gestational age estimates are also useful for quantifying population burdens of preterm birth, which can facilitate appropriate allocation of resources to hospital centres and regions of greatest need. In many low-income and middle-income countries, maternal access to prenatal care, in particular to ultrasound dating services, is limited and imprecise measures, such as last menstrual period, fundal height, or examination of the newborn are relied on for gestational age estimation.^1–3^ Given that gestational dating based on knowledge of last menstrual period is frequently unreliable^4–6^ and postnatal physical examinations are subject to variability based on subjective scoring and poor performance in extreme preterm and SGA infants,^7 8^ new methods of providing gestational age estimates at the time of birth are required. This need has been recognised by organisations who have sought to improve data on preterm birth^9 10^ and also develop new ways to measure gestational age.^11^

In 2013, our research group identified significant variations in newborn screening analyte levels, including amino acid and endocrine markers, based on gestational age.^12^ We subsequently applied these findings to develop a metabolic gestational age algorithm, pairing 3 years of newborn screening data with health administrative information for over 400 000 infants born in Ontario, Canada. The final metabolic postnatal gestational age estimation model consisted of 43 effects including birth weight, sex and a total of 311 model terms. Model performance was evaluated for infants across all categories of gestational age (term, ≥37 weeks; near term, 33–36 weeks; very preterm, 28–32 weeks; extremely preterm, ≤27 weeks), as well as for infants known to be SGA. Our findings demonstrated that this reference model was capable of discriminating between categories of term and preterm birth with robust predictive ability and could also accurately classify infants across a dichotomous preterm birth threshold of 34-weeks gestational age.^13^ The utility of newborn metabolic profiles for postnatal gestational dating has also been confirmed by others.^14 15^

Our work suggests potential value in using dried blood spot-derived analytes for the postnatal assessment of gestational age. This approach may be particularly useful in low-resource settings where reliable estimates of gestational age at the time of birth are difficult to obtain. Postnatal estimates may be useful both in guiding the course of care and for population surveillance of the burden of preterm birth. Before this goal may be realised, however, our previously published gestational age prediction model must be validated in other infant populations. This study aims to evaluate the performance of our reference model on newborn screening profiles taken from infants born in low-resource settings for whom prenatal dating ultrasound scans are available. Additionally, although newborn heel-prick samples are the standard used by newborn screening programs, cord blood may be more easily collected without discomfort to the infant and stress to the parents. We will therefore also evaluate model performance in data derived from cord blood samples.

## Methods/design

### Study objectives

Our primary objective is to use dried blood spot samples collected from Southeast African (Zambian) and South Asian (Bangladeshi) newborns to evaluate the algorithm’s capacity to correctly classify gestational age within 1, 2, 3 and 4 weeks and also within categories of preterm birth.

Our secondary aims are to: 1)determine the accuracy of the reference algorithm in small-for-gestational-age and large-for-gestational-age newborns; 2) determine the ability of the gestational age estimation algorithm to correctly discriminate across dichotomous thresholds of preterm birth (34 weeks, 37 weeks gestational age); and 3) compare the relative performance of algorithms derived from the full newborn screening panel and those restricted to a subset of newborn screening analytes.

### Study design

This project is led by the Ottawa Hospital Research Institute (OHRI) and Newborn Screening Ontario (NSO), located at the Children’s Hospital of Eastern Ontario (CHEO) with oversight of research by the CHEO Research Institute (CHEO-RI), in partnership with the Global Alliance to Prevent Prematurity and Stillbirth (GAPPS), International Centre for Diarrhoeal Disease Research, Bangladesh (icddr,b) and the University of North Carolina, Chapel Hill (UNC-CH) with the University Teaching Hospital (UTH) of Lusaka, Zambia. Funding is provided by the Bill & Melinda Gates Foundation.

This is a non-interventional international validation study to assess the performance of a gestational age estimation model originally developed in a North American population using sex, birth weight and newborn screening values. This protocol outlines the collection, transport and analysis of samples from newborns whose mothers were enrolled into preterm birth cohorts in Lusaka, Zambia and Matlab, Bangladesh. Data handling and reporting procedures are also detailed.

All procedures will be conducted in accordance with local standards of care. This study has been approved by the research ethics boards and institutional review boards of all participating institutes. Research agreements, including but not limited to material and data sharing agreements, will be developed in accordance with the preferred standards of the participating host institutions.

### Study setting

The study population will consist of newborns whose mothers are already enrolled in one of two participating GAPPS-supported birth cohorts: (1) Preterm and Stillbirth Study in Matlab, Bangladesh (PreSSMat Study, icddr,b, Matlab, Bangladesh), a prospective cohort study designed to assess biologic, environmental and social determinants of adverse pregnancy outcomes and (2) Preventing Preterm Birth in Zambia (ZAPPS Study, UNC-CH/UTH, Lusaka, Zambia), a prospective cohort study and biorepository designed to characterise the factors associated with preterm delivery and outcomes of prematurity in Zambia. Sample size requirements for this study are addressed in online supplementary material 1.

10.1136/bmjgh-2017-000365.supp1 Supplementary material 1

### Patient involvement

All participants will provide written informed consent to participate in the study. Enrolment and participation in either the PreSSMat or ZAPPS Study preterm birth cohorts will not be affected by a woman’s choice to participate or not participate in the current protocol. Written consents have been translated into Bangla for participants in Bangladesh and into Bemba and Nyanja for participants in Zambia.

Criteria for enrolment into the proposed study will be those already outlined for the established preterm birth cohorts. For ZAPPS, pregnant women will be enrolled if: ≥18 years old and residing within Lusaka, Zambia; gestational age ≤20 weeks with gestation confirmed by ultrasound; singleton or twin pregnancy with confirmed fetal heart tones; and willing to allow their newborns to participate in the study. For PreSSMat, pregnant women will be enrolled if: >15 years of age and <20 weeks gestation with gestational age confirmed by ultrasound; singleton or twin pregnancy with confirmed fetal heart tones, residing within Matlab, Bangladesh; and willing to participate in all antenatal, delivery and postpartum study milestones. All newborns born into the two cohorts will be eligible for inclusion in the current study. There will be no other explicit inclusion or exclusion criteria.

Consent details already in place for each of the established birth cohorts include provision of information pertaining to the objectives, procedures, risks and benefits to participation. Participants will be made aware that participation is voluntary and that those choosing not to participate will continue to receive antenatal care and treatment according to local clinical standards. Participants will be assured that all reasonable measures will be taken to protect the information of themselves and their newborn. Participants will also consent to storage and future use of their samples for related preterm birth research under authorisation of presiding institutional committees.

Participants will additionally be informed of the risks and benefits of heel-prick blood collection. Participants will be told that heel-prick collection may cause temporary discomfort to their newborn and that through their participation there is a possibility that their child may be identified to be at risk of one or more diseases. Participants will be informed that such incidental findings will be communicated to the study investigators, and recommendations will be made to confirm diagnoses and guide treatment of their infant if necessary. At the time of consent, participants will be informed of their right to request the full details of the screening results for their newborns.

### Sample collection

**Figure 1:**
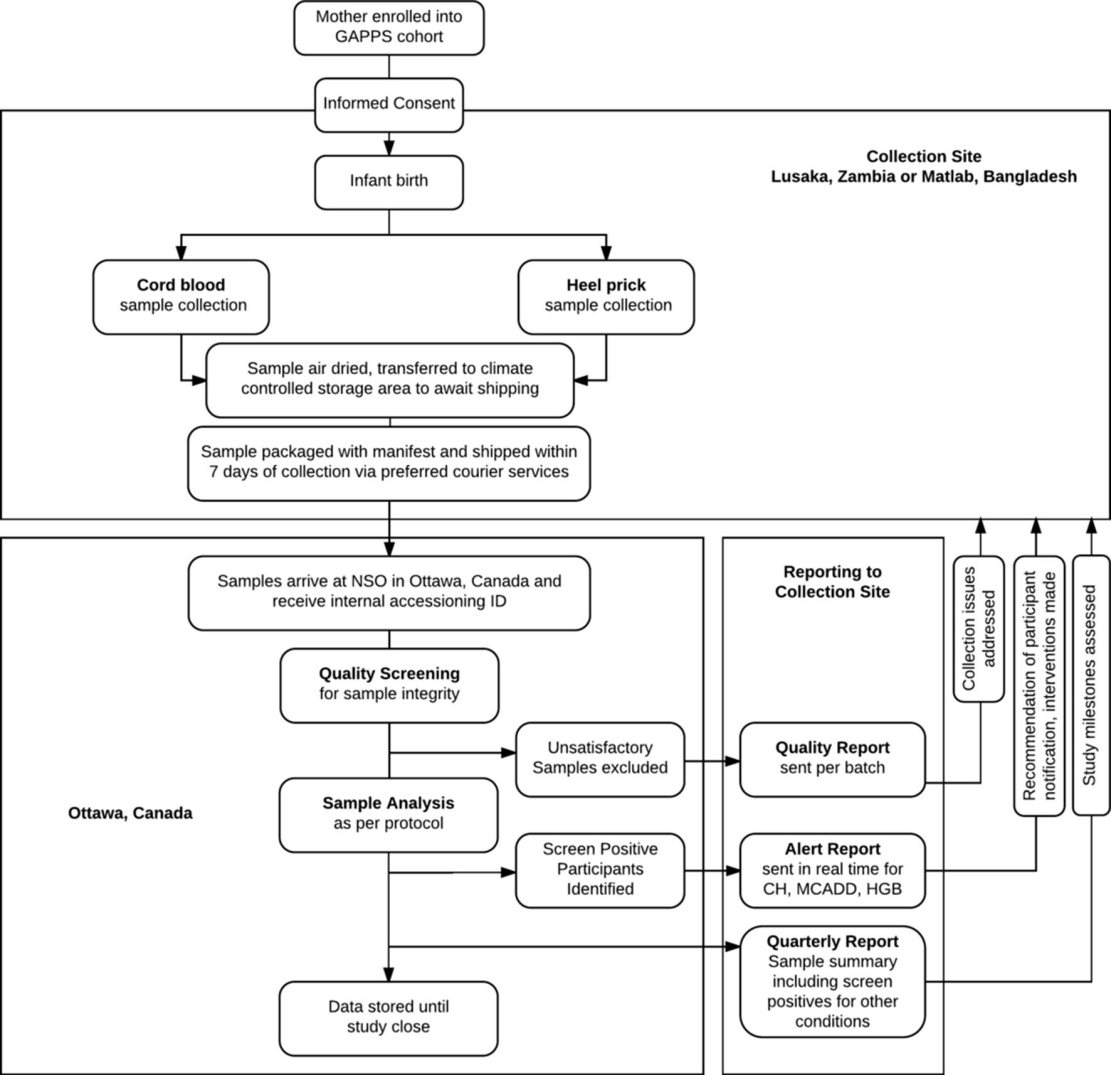
Proposed study workflow. Samples accrued from collection sites in Zambia and Bangladesh will be sent via preferred courier services for analysis at Newborn Screening Ontario (NSO) in Ontario, Canada. Reporting procedures from Ontario, Canada, to the collection sites will include provision of reports on sample quality, on newborns at risk of congenital hypothyroidism (CH), medium-chain acyl-CoA dehydrogenase deficiency (MCADD) and haemoglobinopathies (HGBs), as well as quarterly reports summarising study progress.

Dried blood spot samples will be collected according to standard operating procedures that will be provided to all collection sites. A procedure manual will similarly be distributed to all collection sites detailing the scope of work for all participating institutions, as well as procedures for reporting and management of incidental findings. A summary of the proposed workflow described herein is provided in figure 1.

#### Cord sample collection

Umbilical cord blood or blood from a large placental vein on the chorionic surface will be collected within 30 min of delivery of the placenta into an uncoated, sterile syringe. Drops of blood from the syringe will be applied to designated Whatman 903 filter paper. No more than 400 µL of cord blood will be required for the proposed study.

#### Heel-prick sample collection

To facilitate newborn screening analysis, newborn heel-prick samples will be taken 24–72 hours after birth or prior to discharge if the newborn is released from hospital within 24 hours of delivery. In brief, the newborn’s heel will be warmed at the skin-puncture site to promote increased blood flow. The puncture site will be disinfected and air-dried. A sterile lancet or heel incision device will be used to puncture the lateral aspect of the plantar surface of the newborn's heel. Following heel puncture, the first small drop of blood formed will be cleaned away and the formation of a second large drop of blood will be encouraged by intermittently applying gentle pressure to the newborn’s lower leg and heel. The Whatman 903 filter card will be applied gently against the blood drop and a sufficient quantity of blood allowed to soak through and completely fill a preprinted circle on the filter card. Each preprinted circle on the filter card will be filled by subsequently formed drops of blood. No more than 400 µL of blood will be required for heel-prick sample collection in the proposed study. Following sample collection, the newborn’s foot will be elevated above the body and a sterile gauze pad or cotton swab pressed against the puncture site until the bleeding stops.

Labels with unique cohort-specific de-identified participant IDs and bar codes will be affixed to each filter card at the time of collection. Samples will be air-dried for 3–4 hours following which they will be transported to a designated secure study location (hospital office or laboratory) and stored in a climate-controlled setting prior to shipping. Clinical and demographic information essential for sample analysis will also be captured. Table 1 summarises essential sample information.

**Table 1:** Sample information necessary for newborn screening analysis

Sample information	Comment
Sample ID and bar code number	
Application method to filter card	Direct, tube, syringe
Date and time of birth	Day-month-year; hh:mm
Date and time of sample collection	Day-month-year; hh:mm
Gestational age	Weeks + days
Birth weight	Grams
Sex	Male, female, ambiguous
Multiple birth	Yes, no; if yes, baby 1, 2, 3 or a, b, c, etc
Feeding status	Breast, total parenteral nutrition, formula, nil per os
Packed red blood cell transfusion	Yes, no; if yes, date of latest transfusion
Delivery outcome	Live or stillborn newborn

### Sample shipping and accessioning

Samples will be shipped from collection sites to NSO in Ottawa, Canada, every 7 days via preferred courier services. Appropriate shipping standards will be recommended to the collection sites to minimise risk of compromising sample integrity during the shipping process. A manifest including the sample information summarised in table 1 will be relayed to NSO and OHRI via encrypted electronic documents ahead of shipping and also included in hard copy within the shipment package.

On receipt at NSO, the sample manifest will be cross-referenced against the physical sample cards. Any discrepancies will be resolved through follow-up with the collection site. Secondary accession numbers will be applied for use by internal NSO systems.

### Quality management of samples

#### Quality screening

NSO will refer to organisation and international standards to guide quality management of samples.^16 17^ Each sample received will be reviewed for specimen quality and quantity. A satisfactory newborn screening specimen will have blood fully soaked through to the back of the filter paper; newborn screening test calculations assume that the blood is evenly distributed within the circle and completely saturates both sides of the filter paper.

Unsatisfactory specimens will not be used for study analysis. Specimens may be deemed unsatisfactory for several reasons. Parameters considered by NSO when determining sample quality and project specific actions for analysis are provided in table 2.

**Table 2:** Sample quality screening criteria

Parameter of sample quality	Quality	Action for analysis
Acceptable	Satisfactory	Include
Blood spot collection paper expired	Unsatisfactory	Exclude
Blood spots appear clotted or layered	Unsatisfactory	Exclude
Blood spots appear diluted	Unsatisfactory	Exclude
Blood spots appear scratched or abraded	Unsatisfactory	Exclude
Blood spots appear damaged	Unsatisfactory	Exclude
Blood spots are supersaturated	Unsatisfactory	Exclude
Blood spots are wet/discoloured	Unsatisfactory	Exclude
Blood spots exhibit serum rings	Unsatisfactory	Exclude
Quantity of blood insufficient	Unsatisfactory	Review decision*

*If sample quality is unsatisfactory due to insufficient quantity of blood, the sample will be reviewed at time of receipt and, at the discretion of Newborn Screening Ontario study staff, will be excluded or undergo partial analysis.

Data loggers will be included in each shipment of specimens for assessment of temperature and humidity conditions. Should it be suspected that environmental conditions are affecting sample integrity, temperature and humidity control mechanisms within sample containers will be considered.

#### Quality reporting

NSO will generate reports summarising the quality of samples received on a per-shipment basis. These Quality Reports will be sent electronically to the collection site and used to address issues of sample collection, handling and storage prior to shipment. The Quality Report will summarise the Batch ID for the batch of samples received, temperature and humidity data gathered during the transportation period by data loggers, specimen details and the quality of the samples, as outlined above in table 2.

### Newborn screening analysis

Standard practice at NSO is to screen each sample for metabolites indicative of risk for 29 conditions.^17^ A summary of the newborn screening analytes and analyte ratios available for analysis is provided in table 3.

**Table 3:** Newborn screening analytes

Marker type	Analytes measured
Acyl-carnitines, Acylcarnitine ratios, other	C0, C0|C16, C18, C0, C2, C3, C16, C18|Cit C2 C3, C3DC, C3|C0, C3|C16, C3, C2, C3|C4DC C4, C4OH, C4DC C5, C5:1, C5DC, C5OH, C5|C0, C5|C2, C5|C3, C5DC|C16, C5DC|C5OH, C5DC|C8, C5OH|C2, C5OH|C5:1, C5OH|C8 C6, C6DC C8, C8:1, C8|C10, C8|C2 C10, C10:1 C12, C12:1 C14, C14:1, C14:2, C14:1|C12:1, C14:1|C16, C14.1|C4, C14OH C16, C16OH, C16:1OH C16:1OH|C4DC, C16OH|C16 C18, C18:1, C18:2, C18OH, C18:1OH Medium-chain acyl-CoA
Amino acids, amino acid ratios, other	Alanine, arginine, citrulline, glycine, leucine, methionine, ornithine, phenylalanine, succinylacetone, tyrosine, valine, citrulline:arginine, leucine:alanine, leucine:phenylalanine, methionine:phenlyalanine, phenylalanine:tyrosine, valine:phenylalanine
Cystic fibrosis markers	Immunoreactive trypsinogen (IRT), cystic fibrosis transmembrane conductance regulator gene (*CFTR*) mutation 1, *CFTR* mutation 2, intron 8 polythymidine tract (5T/7T/9T)
Endocrine markers	Thyroid-stimulating hormone, 17-hydroxyprogesterone (17OHP), androstenedione, cortisol
Enzyme markers	Biotinidase (BIOT), galactose-1-phosphate uridyl transferase (GALT)
T-cell function	T-cell receptor excision circles (TRECs)
Haemoglobin variants, haemoglobinopathies and peak percentages	Haemoglobin (HGB): adult haemoglobins, HbA(A) and variants (S, C, D, E, B-thal) HGB peak percentages: HGB-FAST, HGB-F1, HGB-F, HGB-F+F1, HGBFAST+F1, HGB-Other, HGB-A
Purines	Adenosine, deoxyadenosine, guanosine, deoxyguanosine, inosine, deoxyinosine, xanthine, hypoxanthine

Full-panel analyses as per standard NSO practice will be executed on samples when possible (see above: Sample quality screening criteria, Quantity of blood insufficient). Any changes to standard practice of the full-panel analyses will be communicated via electronic bulletins to OHRI and the collection sites. Second-tier analysis of samples, including repeat confirmatory testing, *CFTR* mutation analyses, TBX1 and purine profile assays, will be executed as per standard NSO practice provided sufficient sample is available from the original blood spot card.

Determination of ‘screen positive’ or ‘screen negative’ results will be based on comparison of analyte values against reference ranges set by NSO. Given that alert and screening logic are subject to change, active, complete newborn screening logic will be available to all participating institutions on request.

### Newborn screening results

There are two possible outcomes of newborn screening:

#### Screen negative results

If a newborn infant is screen negative, he or she has a low risk of having any of the diseases included on the screening panel.

#### Screen positive results

If the newborn infant is screen positive, this does not mean that the infant has a disease. However, it does mean that the infant is suspected to be at high risk of having a disease. Screen positive cases will be considered ‘incidental findings’ to the study.

As with all screening tests, false-positive and false-negative results may occur with newborn screening. False-positive results may increase parental anxiety, while false-negative results may give a misleading sense of reassurance. If a newborn infant displays any unfavourable symptoms, the investigators will recommend to each collection site that the child should be investigated regardless of the results of the newborn screen.

### Management of incidental findings

Although the conditions tested as part of the NSO screening panel are extremely rare, it is possible that, in analysing dried blood-spot samples, an infant will be identified to be at high risk of one of the screened conditions. Such risk poses clinical and ethical considerations of gathering newborn screening data for research purposes.

While this study involves newborn screening, this study is *not* being undertaken as a newborn screening initiative and is designed explicitly a non-interventional study. Thus, when considering the management of incidental study findings, it was determined that real-time screening should only be provided for conditions that may be feasibly treated at the collection sites. The following factors were considered when surveying conditions warranting real-time reporting of screen positive cases.

#### Sample integrity

The diagnoses of some newborn screening conditions are affected by the timing of sample collection. Newborn screening samples are ideally collected 24–72 hours after delivery to avoid misdiagnosis as a result of natural postpartum fluctuations in metabolite levels. In this study, it is expected that many mothers will be discharged within 24 hours of birth. Environmental conditions such as heat/humidity may also impact newborn screening results.

#### Potential for confirmatory testing

Ultimately, repeat and more complex confirmatory testing will be required for newborn infants with a screen positive result. Confirmatory testing may require urine analysis, sweat tests, gene sequencing and/or enzyme quantification, the materials and/or expertise for which may be neither available nor affordable to participant families. Conditions for which high positive predictive values could be set or those for which feasible confirmatory tests were available were preferred for real-time reporting.

#### Potential for intervention

The complexity of intervention required to treat identified newborn conditions is an important consideration. Conditions for which standard treatments were simple, inexpensive and effective were preferred for real-time reporting.

Based on the above listed considerations, three high-priority conditions were identified for which real-time reporting of newborn screening results should be provided to the collection sites: congenital hypothyroidism, haemoglobinopathies and medium-chain acyl-CoA dehydrogenase deficiency. In the event that analysis of dried blood-spot samples identifies a newborn infant as screen positive or high risk for any of these three conditions, NSO will circulate individual Alert Reports to the collection sites. In the circulation of Alert Reports, it will be recommended that positive screening results be communicated to patient families and that the newborn receive the appropriate follow-up for confirmatory testing and necessary medical interventions.

### Quarterly reporting

Quarterly Reports summarising study progress will be generated by NSO and shared with the collection site and OHRI. Quarterly Reports will summarise the total number of samples received, sample transport data (minimum, maximum and median time spent in transit, temperature and humidity data), newborn characteristic data (distribution of samples across gestational age categories, sex, multiple births etc) and the number and distribution of samples excluded from analysis based on ‘unsatisfactory’ quality. The Quarterly Report will also include a summary of all screening results of samples received to date.

### Study closure and data exchange

Study samples will be stored in Ottawa, Ontario until study closure, upon which a bulk shipment return of study samples to the collection site via preferred courier services will be accommodated. On return, samples will be stored within designated biorepositories where they will be housed for future investigations into the pathophysiological process of preterm parturition. Recommended storage conditions for dried blood-spot samples are −80°C.

At study closure neonatal, maternal and birth data provided by the collection site (including but not limited to those covariates provided in table 4) will be paired with matching newborn screening data provided by NSO at CHEO-RI into one combined data set and used to complete the study objectives.

**Table 4:** Maternal and neonatal covariates to be included for the study

Neonatal characteristics	Date of ultrasound Gestational age at time of ultrasound (weeks+days) Birth weight (grams) Gestational age (weeks+days) Date and time of birth Date and time of sample collection Feeding status (breast, total parenteral nutrition, formula, nil per os) Packed red blood cell transfusion (yes, no; if yes, date of latest transfusion) Multiple births (yes, no; if yes, baby a, b, c or 1, 2, 3) Low birth weight, intrauterine growth restricted, small- or large-for-gestational age Caesarian section or vaginal delivery (spontaneous or practitioner induced) Presentation at time of delivery Apgar scores
Maternal characteristics	Age (years) Body mass index (kilogram per square metre) Parity and gravidity Smoking status Alcohol consumption (if available) Diabetes Hypertension

## Statistical analyses

Collected data will be used to validate metabolic postnatal gestational age estimation models developed by our group. Specifically, we propose to test four models using methods we have previously described.^18^ While our original published approach included model development based on the full panel of newborn screening analytes, utility of such an algorithm in low-resource settings is likely to be hampered by limited access to advanced laboratory technology, including tandem mass spectrometry (MS/MS). For this reason, we have developed simplified models of gestational age prediction that rely on non-MS/MS-derived analytes and have published the comparative performance of these models in a Canadian cohort.^18^ In brief, we propose to assess the performance of each of the following models: 1) birth weight alone; 2) combination of birth weight and fetal/adult haemoglobin levels; 3) combination of birth weight, haemoglobin levels, thyroid-stimulating hormone and 17-OHP (all non-mass-spectrometry-derived analytes); and 4) birth weight and the full panel of newborn screening analytes. Sex and multiple birth (yes, no) will be included in all models.

### Model validation

All parameter estimates derived in the North American cohort for both the linear and logistic reference models will be fixed at their calculated values and will be used to score the external validation data. Calculated gestational age or the calculated probability of dichotomous prematurity in the logistic setting will be determined. A local slope and intercept will be introduced to linear and logistic models to calibrate the models ‘in the large’.^19^ The residuals will then be calculated for continuous gestational age estimation, as the calculated gestational age minus the actual gestational age, which can in turn be used to calculate root-mean-square error, absolute differences and other performance metrics. For dichotomous prediction, area under the receiver operator curve, sensitivity, specificity and positive and negative predictive values will be derived. The calibration slopes and intercepts will also be reported to describe the calibration correction applied.

### Predictive modelling

Should it be necessary to develop separate models for each of the validation datasets, we will use a multivariable linear regression approach with continuous gestational age in weeks versus newborn screening analytes, sex, multiple birth status (yes, no) and birth weight. Continuous analyte and birth weight values will be modelled using restricted cubic splines. Fetal (F, F1) and adult (A) haemoglobin levels will be modelled as (F+F1)/(A+F+F1) as previously described.^18^ A weighting scheme will be used such that newborns with lower gestational ages will be weighted more heavily for model development, to ensure that term and preterm newborn infants both drive model fitting to a similar degree and to prevent parameter estimates from being overwhelmingly driven by term newborns.

Forward stepwise variable selection will be applied using the *Swartz Bayesian Criterion* to guide the selection of covariates retained in the final model. Models will be internally validated using bootstrap validation methods to address overfitting.^20^

### Model performance for classification as ≤34 weeks or <37 weeks gestational age

We will also develop logistic regression models to distinguish between dichotomous categories of preterm birth (<37 weeks vs ≥37 weeks; ≤34 weeks vs >34 weeks) as we have done previously.^13 18^ The calculated gestational age derived from multiple linear regression models will be used as the independent variable in the logistic regressions, using a restricted cubic spline parameterisation to allow for non-linearity.

### Sensitivity analyses

Model performance in terms of root-mean-square error, absolute prediction within ±1 week, *c*-statistic (area under receiver operator curve) and positive predictive value will be evaluated overall and among newborn infant subgroups (eg, SGA, multiple births) to investigate whether model calculations varied in quality across these subgroups.

## Discussion

This protocol details our approach to the collection and shipment of newborn blood spot samples from international preterm birth cohorts to Ottawa, Canada, for metabolic profiling using newborn screening approaches. We have addressed herein quality management of samples and our analytical approach. The data gathered for this study will be used to validate a postnatal gestational age estimation algorithm originally developed using newborn samples derived from a Canadian cohort.

The strength of our approach includes leveraging the existing framework of large preterm birth cohorts operating in two ethnically distinct populations and our collaboration with NSO, a successful provincial newborn screening programme in Ottawa, Canada, that will lend greatly to the timely and accurate analysis of samples. We acknowledge that successful execution of the study will be dependent on the collection of a satisfactory number of newborn samples from each of the Zambian and Bangladeshi cohorts. A number of prospective participants may be unfamiliar with newborn screening procedures and thus uncomfortable with newborn heel-prick collection. Anticipating that some mothers may choose to decline participation on the basis of the newborn heel prick, participation will be open to those newborns from whom only single, unpaired cord blood or heel-prick samples may be collected. Validation of the metabolic postnatal gestational age estimation model using cord blood profiles will prove equally important to the aims of our study as we seek to optimise the practical utility of our approach. In the event that we find that a single global algorithm is not suitable for all newborn populations, we will tailor our approach to develop region-specific models to enhance gestational age estimation based on specific newborn populations.

As discussed previously, novel methods are warranted to guide both local healthcare interventions and accurate population health estimates for low-resource regions of the world. In addition to providing insight into metabolic variations of newborn infant populations of varying ethnic background, gestational age and condition (eg, low birth weight, SGA), we anticipate that validation of our approach will be of benefit to the search for new reliable methods for estimating gestational age at the time of birth.

## Declarations

### Ethics approval and consent to participate

This study was approved by the Ottawa Health Science Network Research Ethics Board at the OHRI (20160219-01H) and the Research Ethics Board of the Children’s Hospital of Eastern Ontario Research Institute (16/20E). The protocol was also approved by Ethics Review Committee at the icddr,b (PR-16039), in Matlab, Bangladesh, and the University of Zambia’s Biomedical Research Ethics Committee (016-04-14) in Lusaka, Zambia.
