# Supplementary material for: Postnatal gestational age estimation using newborn screening blood spots: a proposed validation protocol
Source: BMJ Glob Health. 2017 Jul 27;2(2):e000365. doi: 10.1136/bmjgh-2017-000365 (PMC5659179; doi:10.1136/bmjgh-2017-000365)
Supplement: Supplementary material 1 [file bmjgh-2017-000365supp001.pdf]

Supplemental Materials

Sample Size

GAPPS has estimated recruiting 2,000 and 4,700 women into the preterm birth collection sites in Zambia and Bangladesh, respectively. Given estimated recruitment rates, and anticipating that some women will decline to participate in the additional requirements of the proposed study, we estimate that a total of 8,000 – 10,000 satisfactory dried blood-spot samples (all heel-prick and cord blood) will be collected.

Preterm birth (birth at <37 weeks gestational age) is reportedly as high as 20% in Bangladesh and 18% at the study site in Zambia, although for our validation exercise we will seek to validate the reference algorithm using both 34 and 37 weeks gestational age as a preterm birth threshold. We acknowledge that model complexity will be limited by the available sample size.

Expanded power calculations have been run to account for preterm birth rates of 2.5%, 5%, 7.5%, 10%, 12.5%, 15%, 17.5% and 20% for each of the collection sites (Appendix A). The area under the receiver operator curve (AUC) is a good measure of discrimination for a logistic regression model that predicts whether a newborn infant is preterm or term. In our reference model, the AUC was 0.99. Given just 1500 samples (either cord blood or heel-prick) from Zambia, there would be reasonable power to detect differences in AUC between validation and reference populations of at least 0.03 for classifying newborns as ≤34 weeks gestational age *versus* >34 weeks gestational age (power 0.63 if the preterm birth rate were 2.5%), and 0.01 for classifying newborns at <37 weeks *versus* ≥37 weeks gestation (power 0.60 if the preterm birth rate were approximately 17.5%) – *e.g.,* the validation population demonstrated an AUC of 0.96 or 0.97 respectively compared to the reference AUC of 0.99. Similarly, if 3,500 participant

samples were obtained from Bangladesh, the study would be well-powered to detect differences of 0.02 for classifying newborns at ≤34 weeks gestational age *versus* >34 weeks gestational age (power 0.66 if the preterm birth rate is approximately 2.5%), and 0.01 for classifying <37 weeks gestation *versus* ≥37 weeks gestational age (power 0.90 assuming a preterm birth rate of approximately 20%).

Although the study may potentially be overpowered with respect to the number of full-term newborns, we will aim to collect and analyze blood-spot samples for all subjects for several reasons. First, secondary analyses will aim to evaluate the performance of the model in small- and large-for-gestational-age term newborns. Standard birthweight and growth curve thresholds have been established in western newborn populations and so it will be important to ascertain distributions for birth weight in both preterm and term births from the study cohorts to determine whether population specific cut-points should be adopted for sensitivity analyses. In addition, if the validation demonstrates that the reference algorithm does not perform well in external populations, we will attempt to develop a population-specific algorithm. Development and validation of region-specific algorithms for samples derived from Zambian and Bangladeshi populations will require data on as many samples as are available in both term and preterm newborns.

Power to detect differences in AUC

**Zambia**							
**Projected Sample Size**	**Estimated Preterm Birth Rate (%)**	**Projected number of term samples**	**Projected number of preterm samples**	**AUC0**	**AUC1**	**Difference**	**Power**
1500	2.5	1462	38	0.99	0.98	-0.01	0.22
1500	2.5	1462	38	0.99	0.97	-0.02	0.45
1500	2.5	1462	38	0.99	0.96	-0.03	0.63
1500	2.5	1462	38	0.99	0.95	-0.04	0.76
1500	2.5	1462	38	0.99	0.9	-0.09	0.98

1500	0.05	1425	75	0.99	0.98	-0.01	0.30
1500	0.05	1425	75	0.99	0.97	-0.02	0.62
1500	0.05	1425	75	0.99	0.96	-0.03	0.81
1500	0.05	1425	75	0.99	0.95	-0.04	0.91
1500	0.05	1425	75	0.99	0.9	-0.09	1.00
1500	7.5	1387	113	0.99	0.98	-0.01	0.38
1500	7.5	1387	113	0.99	0.97	-0.02	0.74
1500	7.5	1387	113	0.99	0.96	-0.03	0.90
1500	7.5	1387	113	0.99	0.95	-0.04	0.97
1500	7.5	1387	113	0.99	0.9	-0.09	1.00
1500	0.1	1350	150	0.99	0.98	-0.01	0.44
1500	0.1	1350	150	0.99	0.97	-0.02	0.81
1500	0.1	1350	150	0.99	0.96	-0.03	0.95
1500	0.1	1350	150	0.99	0.95	-0.04	0.99
1500	0.1	1350	150	0.99	0.9	-0.09	1.00
1500	0.125	1312	188	0.99	0.98	-0.01	0.50
1500	0.125	1312	188	0.99	0.97	-0.02	0.87
1500	0.125	1312	188	0.99	0.96	-0.03	0.98
1500	0.125	1312	188	0.99	0.95	-0.04	1.00
1500	0.125	1312	188	0.99	0.9	-0.09	1.00
1500	0.15	1275	225	0.99	0.98	-0.01	0.55
1500	0.15	1275	225	0.99	0.97	-0.02	0.91
1500	0.15	1275	225	0.99	0.96	-0.03	0.99
1500	0.15	1275	225	0.99	0.95	-0.04	1.00
1500	0.15	1275	225	0.99	0.9	-0.09	1.00
1500	0.175	1237	263	0.99	0.98	-0.01	0.60
1500	0.175	1237	263	0.99	0.97	-0.02	0.94
1500	0.175	1237	263	0.99	0.96	-0.03	0.99
1500	0.175	1237	263	0.99	0.95	-0.04	1.00
1500	0.175	1237	263	0.99	0.9	-0.09	1.00
1500	0.2	1200	300	0.99	0.98	-0.01	0.64
1500	0.2	1200	300	0.99	0.97	-0.02	0.96
1500	0.2	1200	300	0.99	0.96	-0.03	1.00
1500	0.2	1200	300	0.99	0.95	-0.04	1.00

1500	0.2	1200	300	0.99	0.9	-0.09	1.00
**Bangladesh**							
**Projected Sample Size**	**Estimated Preterm Birth Rate (%)**	**Projected number of term samples**	**Projected number of preterm samples**	**AUC0**	**AUC1**	**Difference**	**Power**
3500	2.5	3412	88	0.99	0.98	-0.01	0.33
3500	2.5	3412	88	0.99	0.97	-0.02	0.66
3500	2.5	3412	88	0.99	0.96	-0.03	0.85
3500	2.5	3412	88	0.99	0.95	-0.04	0.94
3500	2.5	3412	88	0.99	0.9	-0.09	1.00
3500	0.05	3325	175	0.99	0.98	-0.01	0.48
3500	0.05	3325	175	0.99	0.97	-0.02	0.86
3500	0.05	3325	175	0.99	0.96	-0.03	0.97
3500	0.05	3325	175	0.99	0.95	-0.04	0.99
3500	0.05	3325	175	0.99	0.9	-0.09	1.00
3500	7.5	3237	263	0.99	0.98	-0.01	0.60
3500	7.5	3237	263	0.99	0.97	-0.02	0.94
3500	7.5	3237	263	0.99	0.96	-0.03	0.99
3500	7.5	3237	263	0.99	0.95	-0.04	0.99
3500	7.5	3237	263	0.99	0.9	-0.09	1.00
3500	0.1	3150	350	0.99	0.98	-0.01	0.69
3500	0.1	3150	350	0.99	0.97	-0.02	0.98
3500	0.1	3150	350	0.99	0.96	-0.03	1.00
3500	0.1	3150	350	0.99	0.95	-0.04	1.00
3500	0.1	3150	350	0.99	0.9	-0.09	1.00
3500	0.125	3062	438	0.99	0.98	-0.01	0.77
3500	0.125	3062	438	0.99	0.97	-0.02	0.99
3500	0.125	3062	438	0.99	0.96	-0.03	1.00
3500	0.125	3062	438	0.99	0.95	-0.04	1.00
3500	0.125	3062	438	0.99	0.9	-0.09	1.00
3500	0.15	2975	525	0.99	0.98	-0.01	0.82
3500	0.15	2975	525	0.99	0.97	-0.02	1.00
3500	0.15	2975	525	0.99	0.96	-0.03	1.00
3500	0.15	2975	525	0.99	0.95	-0.04	1.00
3500	0.15	2975	525	0.99	0.9	-0.09	1.00

3500	0.175	2887	613	0.99	0.98	-0.01	0.87
3500	0.175	2887	613	0.99	0.97	-0.02	1.00
3500	0.175	2887	613	0.99	0.96	-0.03	1.00
3500	0.175	2887	613	0.99	0.95	-0.04	1.00
3500	0.175	2887	613	0.99	0.9	-0.09	1.00
3500	0.2	2800	700	0.99	0.98	-0.01	0.90
3500	0.2	2800	700	0.99	0.97	-0.02	1.00
3500	0.2	2800	700	0.99	0.96	-0.03	1.00
3500	0.2	2800	700	0.99	0.95	-0.04	1.00
3500	0.2	2800	700	0.99	0.9	-0.09	1.00
